# Fasting induces ANGPTL4 and reduces LPL activity in human adipose tissue

**DOI:** 10.1016/j.molmet.2020.101033

**Published:** 2020-06-03

**Authors:** Philip M.M. Ruppert, Charlotte C.J.R. Michielsen, Eric J. Hazebroek, Ali Pirayesh, Gunilla Olivecrona, Lydia A. Afman, Sander Kersten

**Affiliations:** 1 Nutrition, Metabolism and Genomics Group, Division of Human Nutrition and Health, Wageningen University, Wageningen, the Netherlands; 2 Department of Bariatric Surgery, Rijnstate Hospital/Vitalys Clinic, Arnhem, the Netherlands; 3 Nutrition and Disease Group, Division of Human Nutrition and Health, Wageningen University, Wageningen, the Netherlands; 4 Amsterdam Plastic Surgery, Amsterdam, the Netherlands; 5 Department of Medical Biosciences/Physiological Chemistry, Umeå University, Umeå, Sweden

**Keywords:** Adipose tissue, Lipoprotein lipase, Triglycerides, Insulin, Fatty acids, ANGPTL4, Angiopoietin-like 4, ANGPTL8, Angiopoietin-like 8, SVF, Stromal vascular fraction, WAT, White adipose tissue

## Abstract

**Objective:**

Studies in mice have shown that the decrease in lipoprotein lipase (LPL) activity in adipose tissue upon fasting is mediated by induction of the inhibitor ANGPTL4. Here, we aimed to validate this concept in humans by determining the effect of a prolonged fast on ANGPTL4 and LPL gene and protein expression in human subcutaneous adipose tissue.

**Methods:**

Twenty-three volunteers ate a standardized meal at 18.00 h and fasted until 20.00 h the next day. Blood was drawn and periumbilical adipose tissue biopsies were collected 2 h and 26 h after the meal.

**Results:**

Consistent with previous mouse data, LPL activity in human adipose tissue was significantly decreased by fasting (−60%), concurrent with increased ANGPTL4 mRNA (+90%) and decreased ANGPTL8 mRNA (−94%). ANGPTL4 protein levels in adipose tissue were also significantly increased by fasting (+46%), whereas LPL mRNA and protein levels remained unchanged. In agreement with the adipose tissue data, plasma ANGPTL4 levels increased upon fasting (+100%), whereas plasma ANGPTL8 decreased (−79%). Insulin, levels of which significantly decreased upon fasting, downregulated ANGPTL4 mRNA and protein in primary human adipocytes. By contrast, cortisol, levels of which significantly increased upon fasting, upregulated ANGPTL4 mRNA and protein in primary human adipocytes as did fatty acids.

**Conclusion:**

ANGPTL4 levels in human adipose tissue are increased by fasting, likely via increased plasma cortisol and free fatty acids and decreased plasma insulin, resulting in decreased LPL activity.

This clinical trial was registered with identifier NCT03757767.

## Highlights

• 24-h fast in humans reduces LPL activity in subcutaneous adipose tissue.
• 24-h fast in humans increases adipose ANGPTL4 mRNA, protein, and plasma ANGPTL4 levels.
• Cortisol, fatty acids, and insulin regulate ANGPTL4 in vitro.
• ANGPTL4 mediates the reduction in adipose LPL activity during fasting.
• 24-h fast in humans decreases adipose ANGPTL8 mRNA and plasma ANGPTL8 levels.

## 1 Introduction

Elevated plasma triglyceride levels are associated with an elevated risk of atherosclerotic cardiovascular disease [[1], [2], [3], [4]]. Triglycerides are mainly transported in the blood as part of intestine-derived chylomicrons and liver-derived very-low density lipoproteins (VLDLs). The triglycerides in these lipoprotein particles are cleared from the bloodstream through the action of lipoprotein lipase (LPL) [5,6]. Adipocytes and myocytes produce and secrete large amounts of LPL, which is subsequently transported to the luminal side of the capillary endothelium by glycosylphosphatidylinositol-anchored high-density lipoprotein binding protein 1 (GPIHBP1) [[7], [8], [9]]. As a result, mutations in GPIHBP1 or LPL can lead to severe hypertriglyceridemia. In line with the physiological fluctuations in lipid requirement in various tissues, the activity of LPL is highly variable. For example, LPL activity in adipose tissue is decreased by fasting to reduce lipid storage [[10], [11], [12], [13], [14], [15]]. In addition to regulation via changes in LPL gene transcription, LPL activity is primarily controlled at the post-translational level [12,13,16,17]. Key factors involved in post-translational regulation of LPL include fatty acids, which inhibit LPL via product inhibition [18], and the apolipoproteins C1, C2, C3 and A5. In addition, LPL is regulated by three members of the Angiopoietin-like protein family (ANGPTL): ANGPTL3, ANGPTL4 and ANGPTL8 [19].

The current literature places ANGPTL3, ANGPTL4 and ANGPTL8 at the center of the physiological partitioning of circulating triglycerides among various metabolic tissues [19,20]. ANGPTL3 is secreted by the liver as a complex with ANGPTL8 and regulates postprandial LPL activity in adipose tissue and muscle in an endocrine fashion [21,22]. Whereas the production of ANGPTL3 is relatively insensitive to feeding and fasting, the synthesis of ANGPTL8 is highly induced by feeding, which is mediated by insulin [23]. After feeding, the combined action of ANGPTL3/ANGPTL8 reduces the clearance of plasma triglycerides in brown adipose tissue, heart, and muscle, thereby rerouting plasma triglycerides to white adipose tissue and ensuring the replenishment of triglyceride stores [21,22,24,25]. By contrast, ANGPTL4 has emerged as the dominant regulator of LPL activity in the fasted state. Befitting its original name, fasting-induced adipose factor (FIAF), *Angptl4* was cloned as a fasting-induced gene in murine adipose tissue and liver [26]. Subsequent studies demonstrated that ANGPTL4 inhibits LPL activity and raises plasma triglyceride levels in mice [[27], [28], [29]]. Olivecrona found that *Angptl4* mRNA in rat adipose tissue turns over rapidly and that changes in *Angptl4* mRNA expression are inversely correlated to LPL activity, both during the fed-to-fasted and fasted-to-fed transitions [30]. Consistent with a predominant role of ANGPTL4 during fasting, transgenic ANGPTL4 overexpression markedly reduces plasma triglyceride clearance in mice in the fasted but not in the fed state, leading to a reduced uptake of TG-derived fatty acids by numerous tissues such as adipose tissue [31]. Conversely, ANGPTL4 deficiency in mice is associated with enhanced clearance of plasma triglycerides and the uptake of TG-derived fatty acids into adipose tissue in the fasted state [32]. Furthermore, the fasting-induced decrease in adipose tissue LPL activity was abolished in *Angptl4*−/− mice, indicating that ANGPTL4 mediates the repression of LPL activity during fasting [14]. ANGPTL4 inhibits LPL activity by promoting LPL unfolding via direct protein–protein interactions [33]. In mouse adipose tissue, this action of ANGPTL4 triggers LPL cleavage and subsequent degradation [34,35]. The existence of a mechanism regulating LPL degradation/turnover during fasting and requiring the induction of a gene separate from *Lpl* was already suggested prior to the cloning of ANGPTL4 [13,16].

The predominant role of ANGPTL4 in LPL regulation during fasting is likely at least partly related to the upregulation of ANGPTL4 mRNA and protein levels in mouse adipose tissue by fasting [14,26,32,34,35]. In addition, recent evidence suggests that the inhibitory effect of ANGPTL4 on LPL is counteracted by ANGPTL8, levels of which decrease in adipose tissue during fasting [36]. At the whole body level, the upregulation of ANGPTL4 during fasting ensures that triglycerides are directed to non-adipose tissues to be used as fuel rather than being stored. The importance of ANGPTL4 in the regulation of human plasma triglyceride metabolism is supported by human genetic studies, which have shown that carriers of the E40K mutation and other inactivating variants have reduced plasma triglyceride concentrations and a reduced risk of coronary artery disease [37,38]. The crucial role of ANGPTL4 in governing plasma lipid levels in mice and humans has made ANGPTL4 an attractive therapeutic target for correcting dyslipidemia and associated cardiovascular disorders.

While there is overwhelming support for the role of ANGPTL4 as a fasting-induced inhibitor of LPL activity in rodent adipose tissue, evidence on ANGPTL4 in human adipose tissue is lacking. We have previously shown that human plasma ANGPTL4 levels increase with caloric restriction and during extended fasting [39] and that tissue ANGPTL4 and LPL protein levels negatively correlate in a cross-sectional analysis of human adipose tissue samples [40]. However, whether fasting influences ANGPTL4 protein levels and LPL activity in human adipose tissue remains unclear. Accordingly, the primary objective of this study is to determine the effect of a prolonged fast on ANGPTL4 gene and protein expression in human subcutaneous adipose tissue. An additional aim is to study the effect of a prolonged fast on LPL gene expression, LPL protein expression, and on LPL activity in subcutaneous adipose tissue. To characterize the mechanisms for the regulation of ANGPTL4 by fasting in human adipose tissue, we performed in vitro studies using primary human adipocytes.

## 2 Materials and methods

### 2.1 FASTING study

The FASTING study was approved by the Medical Ethics Committee of Wageningen University and registered at ClinicalTrials.gov, identifier: NCT03757767. In short, 24 healthy volunteers aged 40–70 years (median age 55 years) with a BMI of 22–30 kg/m^2^ (median BMI 25 kg/m^2^) were asked to consume a standardized meal until full (ad libitum), consisting of 22 energy% protein, 24 energy% fat, 51 energy% carbohydrate and 476 kJ per 100 g. Two hours after consumption of the standardized meal, blood samples and a subcutaneous adipose tissue biopsy were taken. Twenty-four hours later, a second subcutaneous adipose tissue biopsy was taken and again blood samples were drawn. After consumption of the standardized meal until after the second measurements, subjects were only allowed to drink water. The subcutaneous adipose tissue samples were obtained by needle biopsy from the periumbilical area under local anesthesia. The samples were rinsed to eliminate blood and were immediately frozen in liquid nitrogen. All samples were stored in aliquots at −80 °C.

### 2.2 Isolation and differentiation of human stromal vascular fraction

Anonymous samples of subcutaneous and visceral adipose tissue were collected from the abdominal region of patients undergoing elective cosmetic surgery at the Amsterdam Plastic Surgery, Amsterdam, The Netherlands (subcutaneous) or bariatric surgery for weight management at the Department of Bariatric Surgery, Rijnstate Hospital/Vitalys Clinic, Arnhem, The Netherlands (visceral). All study subjects gave written informed consent for the use of the tissue.

Material was collected in DMEM supplemented with 1% PS and 1% bovine serum albumin (BSA; Sigma–Aldrich). Upon arrival in the lab, the material was minced with scissors immediately and digested in collagenase-containing medium (DMEM with 3.2 mM CaCl2, 1.5 mg/mL collagenase type II (C6885, Sigma–Aldrich), 10% FBS, 0.5% BSA, and 15 mM HEPES) for 45–60 min at 37 °C, with occasional vortexing. Cells were filtered through a 100-μm cell strainer (Falcon) to remove remaining cell clumps and lymph nodes. The cell suspension subsequently was centrifuged at 1600 rpm for 10 min and the pellet was resuspended in erythrocyte lysis buffer (155 mM NH_4_Cl, 12 mM NaHCO_3_, 0.1 mM EDTA). Upon incubation for 2 min at room temperature, cells were centrifuged at 1200 rpm for 5 min and the pelleted cells were resuspended in Growth medium (DMEM + 10% FBS + 1% P/S) and plated.

Upon confluence, the stromal vascular fraction (SVF) from subcutaneous origin were differentiated according to the standard protocol for 3T3-L1 cells with addition of 1 μM rosiglitazone [41]. Briefly, confluent SVFs were plated in 1:1 surface ratio, and differentiation was induced 2 days later by switching to a differentiation induction cocktail (DMEM containing 10% FBS, 1% P/S, 0.5 mM isobutylmethylxanthine, 1 μM dexamethasone, 7 μg/mL insulin and 1 μM rosiglitazone) for 3 days. Subsequently, cells were maintained in Growth medium with addition of 7 μg/mL insulin for 3–6 days and switched to Growth medium only for 3 days, after which experiments have been performed. The average rate of differentiation was at least 80% as determined visually.

SVFs from visceral origin were differentiated according to the 3D protocol described by Emont et al. [42] due to the very low differentiation rate of visceral preadipocytes when differentiated in a 2D well format. Briefly, pre-adipocytes were seeded at a concentration of 300,000 cells/500 μL collagen gel in a 24-well plate format. Pre-adipocytes were resuspended in Growth medium to a concentration of 6 × 10^6^ cells/mL, of which, per well, 50 μL were mixed with 100 μL 5x DMEM (Biozol, #1–25K34-I), 50 μL FBS and 50 μL 0.1 N NaOH and 250 μL collagen solution (Corning, #354249), in this order, to create the 3D gel. After each step, the solution was carefully mixed by pipetting up and down. The collagen solution was previously diluted to 8 mg/mL with 0.02 N Acetic acid. The 3D gel was allowed to polymerize for 10–20 min in the incubator after which 0.5 mL of growth medium were added per well. Differentiation was induced the next day according to the protocol described for the differentiation of subcutaneous pre-adipocytes. All cells were maintained in a humidified incubator at 37 °C with 5% CO_2_.

### 2.3 Cell culture treatments

Treatments of primary cells were done within 2 days after reaching differentiation. Cells were maintained in DMEM containing 10% FBS and 1% P/S until treatment with insulin (500 nM), cortisol (1 μM), dexamethasone (1 μM), or a mixture of oleate and palmitate (2:1, 300 μM total) for 24 h. In a separate experiment, primary cells were incubated with 40 μg/mL cycloheximide for indicated durations. All compounds were from Sigma–Aldrich.

### 2.4 RNA isolation & quantitative real-time PCR

Total RNA from subcutaneous adipose tissue from the FASTING study was isolated using TRIzol reagent (Thermo Fisher Scientific, the Netherlands) and purified using the Qiagen RNeasy Mini kit (Qiagen, the Netherlands). Total RNA from in vitro studies was isolated homogenizing in TRIzol (Thermo Fisher Scientific) either with a Qiagen Tissue Lyser II (Qiagen, Venlo, The Netherlands) (visceral) or by pipetting up and down (subcutaneous). Reverse transcription was performed using the iScript™ cDNA Synthesis Kit (Biorad, the Netherlands) according to the manufacturer's protocol using 400–750 ng RNA for in vitro studies and 350 ng from human adipose tissue. Quantitative PCR amplifications were done on a CFX 384 Bio-Rad thermal cycler (Bio-Rad, the Netherlands) using SensiMix PCR reagents (Bioline, GC Biotech, the Netherlands). Gene expression values were normalized to one of the housekeeping genes. Primer sequences of genes are provided in Supplemental Table 1.

### 2.5 Western blots

Protein lysates were prepared from subcutaneous adipose tissue from participants included in the FASTING study. The material was lysed in a RIPA lysis buffer (25 mM Tris–HCl pH 7.6, 150 mM NaCl, 1% NP-40, 1% sodium; deoxycholate, 0.1% SDS; Thermo Fisher Scientific, the Netherlands) and supplemented with protease and phosphatase inhibitors (Roche, The Netherlands) to make 20% protein lysates. After a 30-minute incubation on ice, the lysates were spun down at 13.000 rpm at 4 °C for 15 min in order to eliminate non-dissolved material and fat. Following the transfer of the infranatant to a clean tube, this procedure was repeated twice to get rid of excess fat. Protein concentrations of lysates were determined with BCA reagent (Thermo Fisher Scientific, the Netherlands). Next, lysates were mixed with 4x LSB loading buffer and denatured at 95 °C for 5 min. For each participant, 10 μg of protein was loaded per lane on 26- wells Criterion 8–16% TGX gels (Bio-Rad, the Netherlands) and separated by SDS gel electrophoresis. Separated proteins were transferred to a PVDF membrane by means of a Transblot Turbo System (Bio-Rad, the Netherlands). Primary antibodies goat anti-human LPL antibody (Santa Cruz Biotechnology, #Y-20) and rabbit anti-human ANGPTL4 antibody (1187) [43] were used at a ratio of 1:1000 (#Y-20) or 1:5000 (1187). Rabbit anti-human GAPDH was used at 1:2000 (Cell signaling, #2118). All primary antibodies were incubated overnight at 4 °C. Corresponding secondary antibodies (HRP-conjugated) (Sigma–Aldrich, the Netherlands) were used at a 1:5000 dilution. All incubations were done in Tris-buffered saline, pH 7.5, with 0.1% Tween-20 (TBS-T) and 5% dry milk, whereas all washing steps were done in TBS-T without dry milk. Blots were visualized using the ChemiDoc MP system and Clarity ECL substrate (Bio-Rad, the Netherlands). The quantification of bands was performed using ImageLab software (Bio-Rad, the Netherlands).

### 2.6 Quantification of plasma parameters

Blood samples were collected in EDTA-coated tubes and centrifuged at 4 °C for 15 min at 10,000 g. Plasma was collected and stored at −80 °C. Measurements of plasma levels of non-esterified fatty acids (NEFA) and beta-hydroxybutyrate were performed using kits from WAKO Diagnostics (Cat: 3055 and 417-73501/413-73601, WAKO Diagnostics, Germany) according to the manufacturer's protocol. Glucose, insulin, and triglycerides as well as total-, HDL-, and LDL-cholesterol were determined in lithium heparin plasma samples by the hospital Gelderse Vallei, Ede, The Netherlands.

Plasma ANGPTL4 concentrations were determined using the ELISA kit from R&D systems (Cat: DY3485, R&D systems, the Netherlands) according to the manufacturer's protocol. Plasma Cortisol concentrations were determined using the ELISA kit from R&D systems (Cat: KGE008B R&D systems, the Netherlands) according to the manufacturer's protocol.

Plasma ANGPTL8 concentrations were determined by a sandwich ELISA assay using two monoclonal antibodies: a capture antibody to the N-terminal domain and detection antibody to the C-terminal domain (Hobbs, manuscript in preparation).

### 2.7 LPL activity measurements

Frozen subcutaneous adipose tissues biopsies were homogenized in 9 volumes of buffer at pH 8.2 containing 0.025 M ammonia, 1% Triton X-100, 0.1% SDS and protease inhibitor cocktail tablets (Complete Mini, Roche Diagnosis, Germany) using a Polytron PT 3000 Homogenizer (Kinematica). The homogenates were centrifuged for 15 min at 10,000 rpm, 4 °C. Aliquots of the supernatants were used for determination of LPL activity as previously described using a phospholipid-stabilized emulsion of soy bean triacylglycerols and 3H-oleic acid-labeled triolein with the same composition as Intralipid 10% (Fresenius Kabi, Uppsala, Sweden) [13]. The incubation was at 25 °C for 100 or 120 min. One milliunit of enzyme activity corresponds to 1 nmol of fatty acids released per minute. Enzyme activity is expressed per g wet tissue weight. Protein contents in homogenates of adipose tissue were measured using Markwell's modified Lowry method [44].

### 2.8 Statistical analyses

Differences in plasma parameters, LPL activity, and subcutaneous adipose tissue gene expression between the fed and fasted state were evaluated using a paired Student's *t*-test. Individuals are always represented by the same color in the various fed-fasted line graphs. Differences in gene expression in the primary adipocytes were evaluated by unpaired Student's *t*-test. P-values < 0.05 were considered statistically significant.

## 3 Results

**Figure 1:**
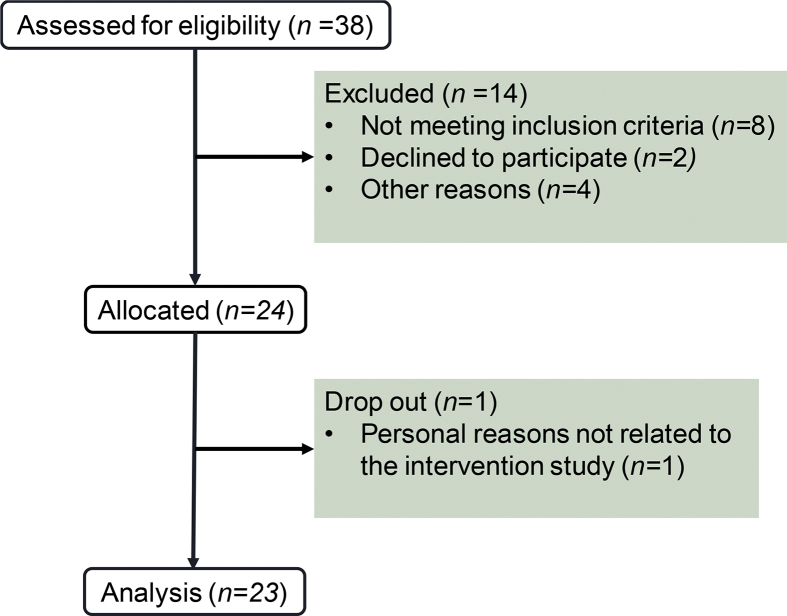
Flow chart of the FASTING study.

Between October and December 2018, 38 individuals were assessed for eligibility of which 14 were excluded from participation (Figure 1). The remaining 24 participants were invited to the research facilities for the FASTING study. One participant dropped out of the study, due to personal reasons that were not related to the study. The remaining 23 participants completed the study.

The main objective of this study was to determine the effect of a prolonged fast on ANGPTL4 gene and protein expression in human subcutaneous adipose tissue and to link these effects with changes in LPL expression and activity. The 23 healthy middle-aged men and women underwent a 24-hour fast. Participants received a standardized meal at 18.00 h, followed by blood sampling and collection of an adipose tissue biopsy at 20.00 h, representing the fed state. At 20.00 h the next day, a second blood sample and adipose tissue biopsy were collected. Accordingly, the two blood samples and adipose tissue biopsies were taken at the same time, thereby avoiding the potential influence of circadian rhythmicity. The participant characteristics are listed in Table 1.

**Table 1:** Participants characteristics.

Participants, n	24
Gender, n males (%)	8 (33%)
Age, years	43–71
Weight, kg	76.5 ± 10.5
BMI, kg/m^2^	25.3 ± 2.4
Plasma cholesterol, mM	5.76 ± 0.96
HDL cholesterol, mM	1.68 ± 0.32
LDL cholesterol, mM	3.02 ± 0.84

**Figure 2:**
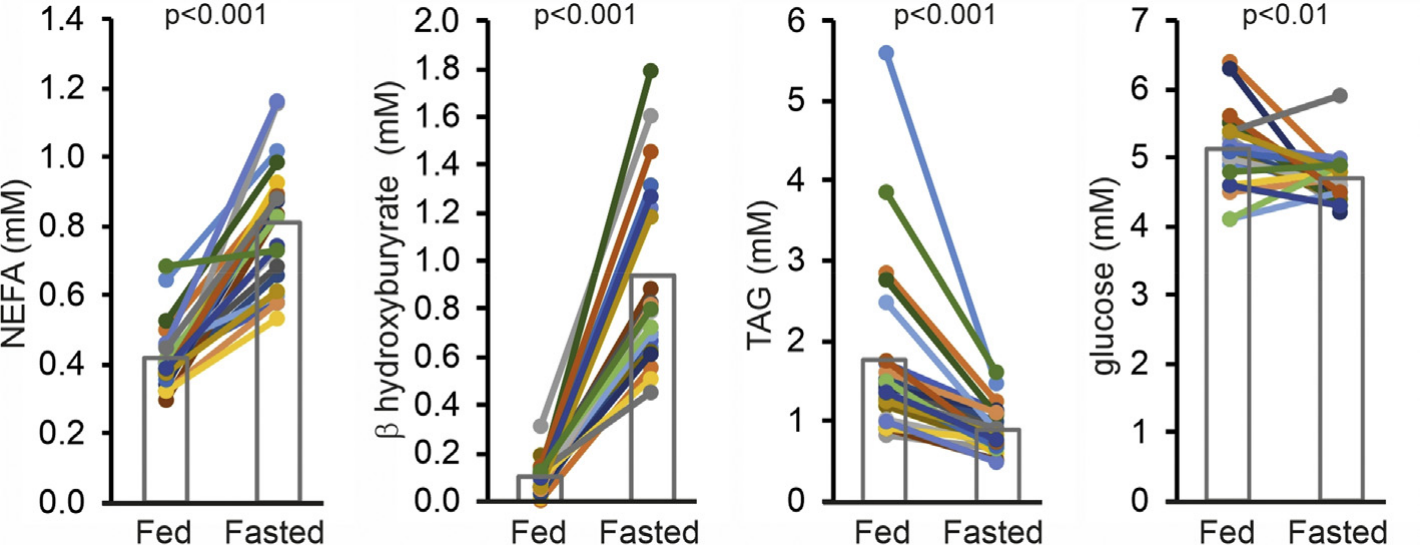
Influence of fasting on plasma metabolites. Plasma concentrations of non-esterified fatty acids (NEFA), β-hydroxybutyrate, triacylglycerol (TAG) and glucose after 2 h (Fed) and 26 h (Fasted) of fasting. Each line represents one individual. Individuals are depicted in the same color in all figures. Bars represent group means (N = 23).

Fasting significantly increased plasma levels of non-esterified fatty acids and β-hydroxybutyrate and significantly decreased plasma triglycerides (Figure 2). The direction of the change is consistent with the known stimulatory effect of fasting on adipose tissue lipolysis and hepatic ketogenesis and the inhibitory effect on plasma triglyceride secretion. Plasma glucose levels showed a more mixed response with most individuals showing a decrease, while six individuals showed an increase (Figure 2). Overall, these parameters confirm compliance with the fasting protocol.

**Figure 3:**
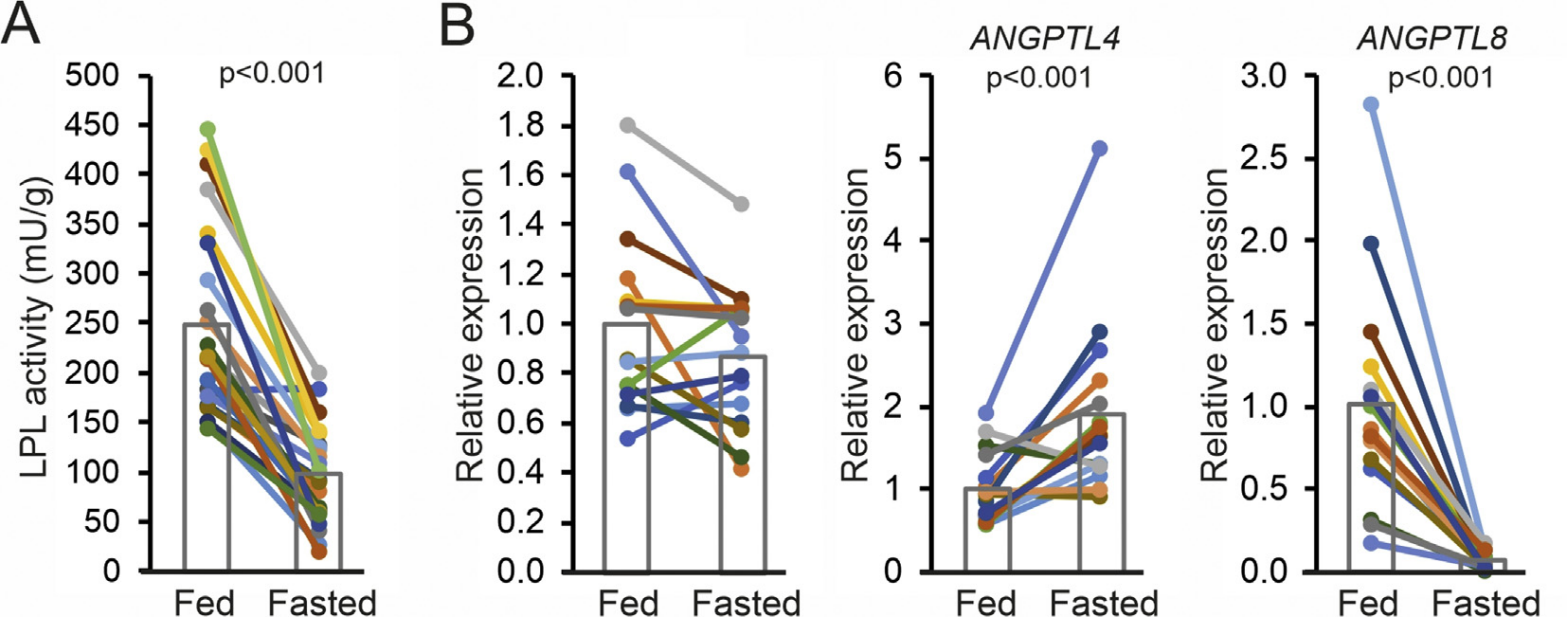
Fasting reduces LPL activity and increases *ANGPTL4* mRNA expression in human adipose tissue biopsies. A) LPL activity measured in subcutaneous adipose tissue samples collected after 2 h (Fed) and 26 h (Fasted) of fasting. Every line represents one individual and bars represent group means (N = 23). B) Relative mRNA levels for *LPL*, *ANGPTL4* and *ANGPTL8* in subcutaneous adipose tissue biopsies collected after 2 h (Fed) and 26 h (Fasted) of fasting, as determined by qPCR. Each line represents one individual and bars represent group means (N = 16). Individuals are depicted in the same color in all figures. The lower number of samples is due to limited availability of biopsy material. Statistical differences were assessed using the paired Student's *t*-test.

In agreement with data from rats and mice [14], fasting led to a marked decrease in adipose tissue LPL activity (−60%, p < 0.001) (Figure 3A), which was consistently observed in all participants. To determine the potential cause of the decrease in LPL activity, we measured the mRNA levels of LPL, ANGPTL4 and ANGPTL8 by qPCR in a subsection of the participants. LPL mRNA was slightly lower after the 24-hour fast but the difference did not reach statistical significance (Figure 3B). In most but not all individuals, adipose ANGPTL4 mRNA levels went up by fasting, with a mean increase of nearly two-fold (+90%, p < 0.001). By contrast, ANGPTL8 mRNA levels went down drastically by fasting (−94%, p < 0.001) (Figure 3B).

**Figure 4:**
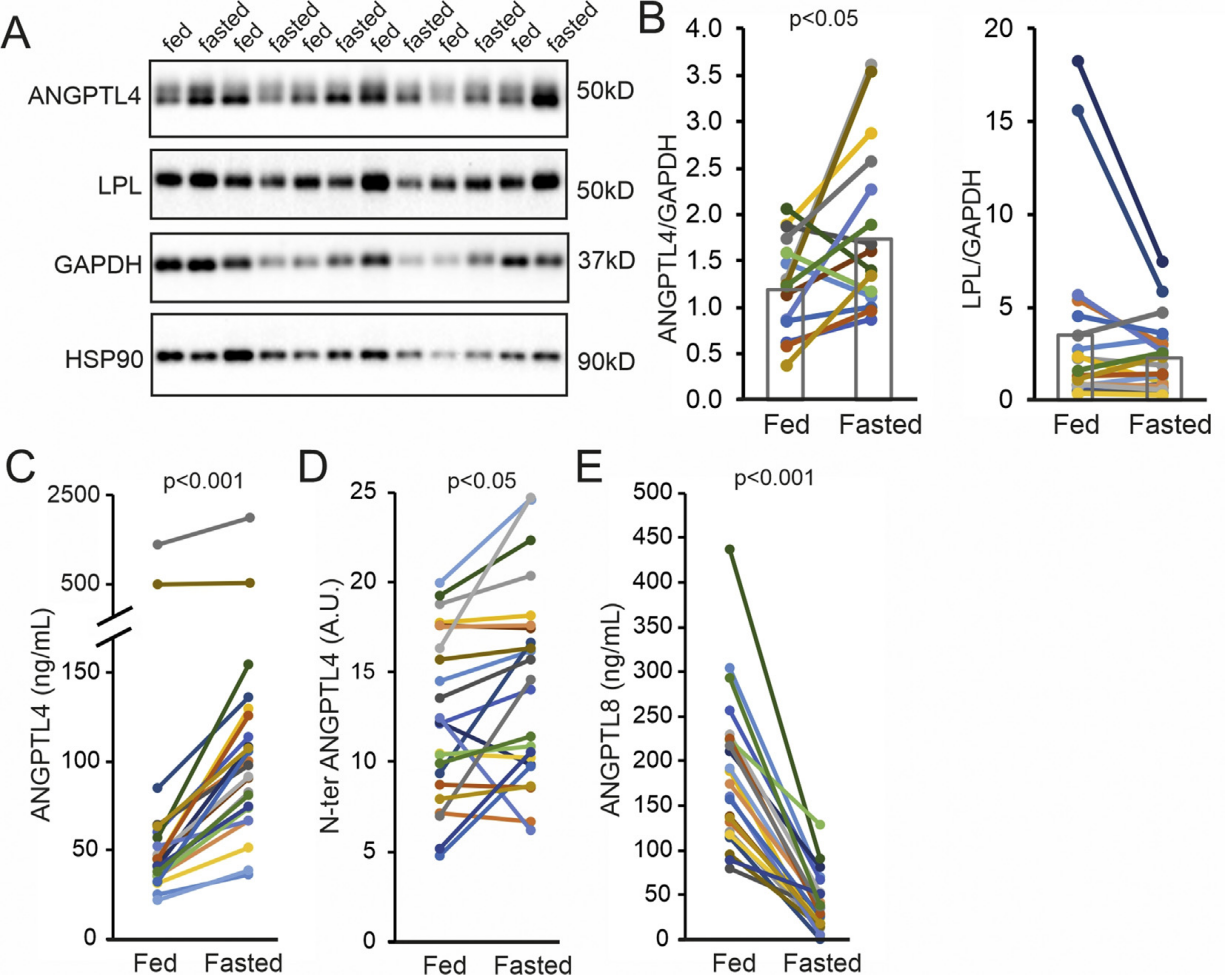
Fasting increases ANGPTL4 protein in human adipose tissue and plasma. A) Representative Western blots for ANGPTL4, LPL, GAPDH and HSP90 in subcutaneous adipose tissue samples of six individuals collected after 2 h (Fed) and 26 h (Fasted) of fasting. B) Quantitative analysis of ANGPTL4 and LPL protein levels in subcutaneous adipose tissue normalized to GAPDH. Each line represents one individual and bars representing group means (N = 19 resp. 20). C) Plasma levels of ANGPTL4 after 2 h (Fed) and 26 h (Fasted) of fasting as determined by ELISA (N = 23). D) Plasma levels of N-terminal ANGPTL4 after 2 h (Fed) and 26 h (Fasted) of fasting as determined by western blot (N = 23). E) Plasma levels of ANGPTL8 after 2 h (Fed) and 26 h (Fasted) of fasting as determined by ELISA (N = 23). Individuals are depicted in the same color in all figures.

To determine whether the increase in ANGPTL4 mRNA by fasting was accompanied by an increase in ANGPTL4 protein, we assessed ANGPTL4 protein levels in the subcutaneous adipose tissue biopsies using Western blot. As shown previously [43], only full length ANGPTL4 was detectable in human adipose tissue, which is in contrast to human liver where we observed substantial N-terminal ANGPTL4 cleavage product (Supplemental Fig. 1A). In most but not all individuals, the level of full length ANGPTL4 protein increased upon fasting (+46%, p < 0.05). Conversely, the mean LPL protein level was lower after fasting, although this decrease did not reach statistical significance (Figure 4A,B). To determine whether changes in ANGPTL4 mRNA and protein levels were associated with changes in plasma ANGPTL4 levels, we measured the plasma ANGPTL4 concentration before and after fasting using ELISA. In agreement with previous data [39], plasma ANGPTL4 concentrations went up in all participants (+100%, p < 0.001) (Figure 4C). As the ANGPTL4 ELISA only measures full length and C-terminal ANGPTL4 [45], we also determined plasma levels of N-terminal ANGPTL4 by Western blot. Fasting modestly yet significantly induced plasma N-terminal ANGPTL4 levels (+15%, p < 0.05) (Figure 4D). By contrast, plasma ANGPTL8 concentrations were decreased by fasting in all participants (−79%, p < 0.001) (Figure 4E).

**Figure 5:**
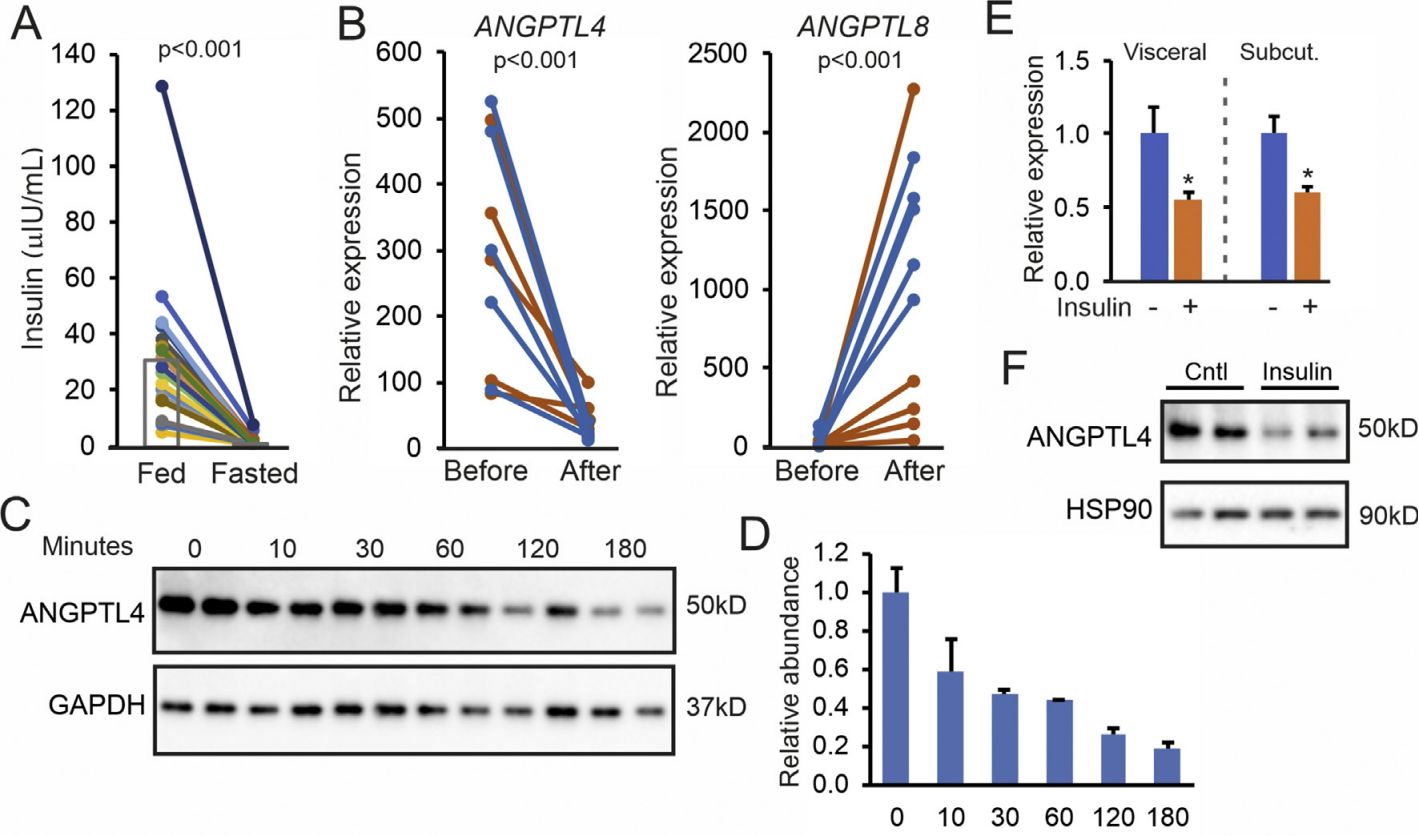
Insulin downregulates ANGPTL4 in vivo and in vitro. A) Plasma insulin concentration after 2 h (Fed) and 26 h (Fasted) of fasting. Each line represents one individual. Individuals are depicted in the same color in all figures. Bars represent group means (N = 23). B) Adipose tissue mRNA levels of *ANGPTL4* and *ANGPTL8* in insulin-sensitive (blue lines) and insulin-resistant (orange lines) subjects before and after a hyperinsulinemic clamp. Data were extracted from GSE26637. C) Western blot for ANGPTL4 and GAPDH in primary human subcutaneous adipocytes treated with cycloheximide for different durations. D) Quantification of the ANGPTL4 levels relative to GAPDH. E) ANGPTL4 mRNA in primary human visceral and subcutaneous adipocytes treated with 500 nM insulin for 24 h. F) Western blot for ANGPTL4 and HSP90 in primary human visceral adipocytes treated with insulin. HSP90 was blotted as a loading control. Statistical differences for in vitro experiments were assessed using the unpaired Student's *t*-test. ∗p < 0.05, relative to control treatment.

The expression of ANGPTL4 in adipose tissue of mice is known to be under the control of various stimuli and transcriptional regulators. For instance, ANGPTL4 expression was previously found to be repressed by insulin [46]. In our study, fasting drastically reduced plasma levels of insulin (−95%, p < 0.001) (Figure 5A), suggesting the increase in adipose ANGPTL4 levels during fasting might be related to the decline in plasma insulin. To investigate whether insulin lowers ANGPTL4 expression in vivo, we extracted data from a transcriptomics dataset of adipose tissue biopsies from human subjects before and after 3 h intravenously maintained euglycemic hyperinsulinemia [47]. Strikingly, ANGPTL4 mRNA levels were markedly reduced by insulin in vivo in both insulin-sensitive and resistant individuals (Figure 5B). By contrast, ANGPTL8 mRNA levels increased, especially in the insulin-sensitive individuals (Figure 5B). These data support a possible role of insulin in the regulation of ANGPTL4 expression in human adipose tissue during fasting but do not indicate whether insulin has a direct role in regulating ANGPTL4. To address this question, we cultured primary human adipocytes. In these cells, full length ANGPTL4 protein was easily detectable by immunoblot, whereas N-terminal ANGPTL4 was absent (Supplemental Fig. 1B). The lack of ANGPTL4 cleavage in cultured human adipocytes is supported by experiments in human Lisa-2 adipocytes (Supplemental Fig. 1C) and SGBS adipocytes [43,48]. Levels of ANGPTL4 protein declined rapidly after treatment with cycloheximide, indicating that ANGPTL4 has a fast turnover in human adipocytes (Figure 5C,D).

**Figure 6:**
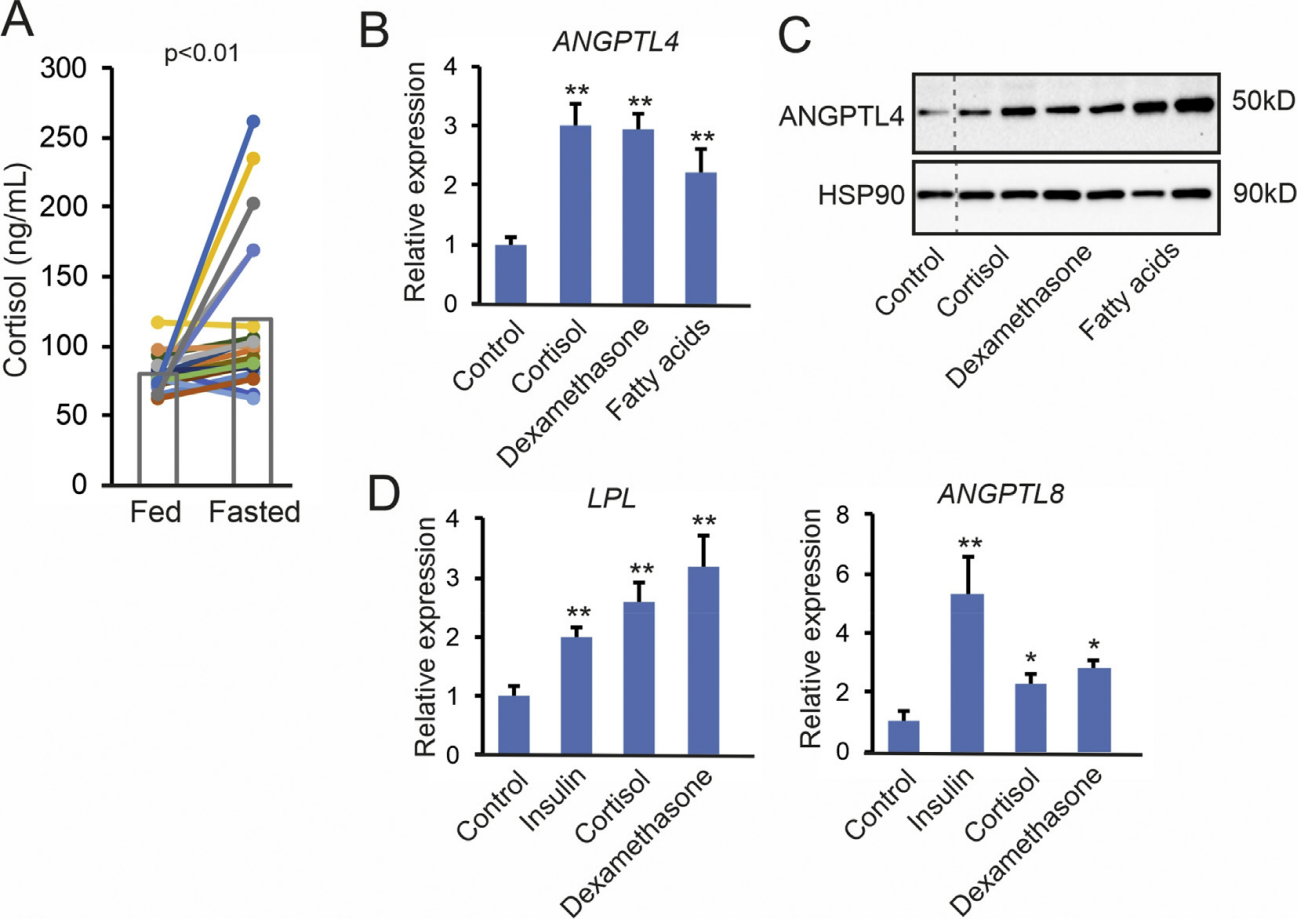
Corticosteroids and fatty acids upregulate ANGPTL4 in primary human adipocytes. (A) Plasma cortisol concentration after 2 h (Fed) and 26 h (Fasted) of fasting. Each line represents one individual. Individuals are depicted in the same color in all figures. Bars represent group means (N = 23). *ANGPTL4* mRNA (B) and ANGPTL4 protein levels (C) in primary human visceral adipocytes treated with cortisol (1 μM), dexamethasone (1 μM), or a mixture of oleate and palmitate (2:1, 300 μM total) for 24 h. HSP90 was blotted as a loading control. D) *LPL* and *ANGPTL8* mRNA in primary human subcutaneous adipocytes treated with insulin (500 nM), cortisol (1 μM), or dexamethasone (1 μM) for 24 h. Statistical differences for in vitro experiments were assessed using the unpaired Student's *t*-test. ∗p < 0.05, ∗∗p < 0.01, ∗∗∗p < 0.001, relative to control treatment.

In primary adipocytes from visceral and subcutaneous adipose tissue, insulin significantly reduced *ANGPTL4* mRNA after the 24-hour treatment (Figure 5E). In visceral adipocytes, insulin also markedly decreased ANGPTL4 protein levels (Figure 5F). In line with data from mouse adipocytes [35,46], these data indicate the insulin directly suppresses ANGPTL4 gene and protein expression in human adipocytes. Besides insulin, another factor that may be involved in regulating ANGPTL4 levels during fasting is cortisol. Fasting significantly increased plasma cortisol concentrations in the human volunteers (Figure 6A). In the primary human adipocytes, cortisol as well as dexamethasone significantly increased ANGPTL4 mRNA and protein levels (Figure 6B). Interestingly, free fatty acids, the plasma levels of which were elevated during fasting (Figure 2), also increased ANGPTL4 mRNA and protein levels (Figure 6B). These data suggest that the increase in ANGPTL4 production in adipose tissue upon fasting is likely mediated by increased plasma cortisol and free fatty acids as well as by decreased plasma insulin.

Consistent with the increase in *ANGPTL8* mRNA levels in adipose tissue after insulin infusion, insulin markedly increased *ANGPTL8* mRNA levels in primary human adipocytes (Figure 6C). Cortisol and dexamethasone also induced *ANGPTL8* mRNA but to a smaller extent. Intriguingly, *LPL* mRNA levels in the primary human adipocytes were significantly increased by insulin, cortisol, and dexamethasone (Figure 6C).

As ANGPTL4 and ANGPTL8 are regulated by insulin, we hypothesized that *ANGPTL4* and *ANGPTL8* mRNA levels in human adipose tissue may respond to weight loss, which is known to increase insulin sensitivity. We analyzed adipose gene expression data from subjects before and after 5 weeks on a very-low-calorie diet (500 Kcal/day), followed by a 4-week weight maintenance diet based on their individual energy requirements [49]. Intriguingly, after 5 weeks of a very-low-calorie diet, when subjects were in a hypocaloric state and actively losing weight, *ANGPTL4* and *ANGPTL8* mRNA levels were significantly increased and decreased, respectively (Supplemental Fig. 2). By contrast, after weight loss and in a eucaloric state of weight stability, *ANGPTL4* and *ANGPTL8* mRNA had returned to pre-weight loss values. *LPL* mRNA largely followed *ANGPTL8* mRNA. These data indicate that *ANGPTL4*, *ANGPTL8*, and *LPL* mRNA in human adipose tissue are not affected by weight loss as such but respond to a negative energy balance.

## 4 Discussion

In this paper we show that a 24-hour fast in human volunteers markedly reduces LPL activity in subcutaneous adipose tissue, concomitant with significant increases in adipose tissue *ANGPTL4* mRNA, adipose tissue ANGPTL4 protein, and plasma ANGPTL4 levels. By contrast, fasting decreases adipose tissue *ANGPTL8* mRNA and plasma ANGPTL8 levels. In cultured human adipocytes, insulin significantly decreased ANGPTL4 mRNA and protein, whereas cortisol and fatty acids had the opposite effect. Inasmuch as plasma insulin levels decrease upon fasting, and plasma cortisol and free fatty acid levels increase upon fasting, the increase in ANGPTL4 production in adipose tissue upon fasting is likely mediated by changes in these factors. Overall, our results strongly support the notion derived from studies in rodents that local upregulation of ANGPTL4 mediates the decrease in LPL activity and associated lipid storage in adipose tissue during fasting in humans. Consistent with its role as rapidly inducible regulator of LPL activity during fasting, we found that ANGPTL4 protein in human adipocytes turns over rapidly, at a rate that is faster than the turnover rate of LPL protein and activity determined in rat adipose tissue [13,14].

LPL activity controls plasma triglyceride clearance [6]. The activity of LPL is differentially regulated in various tissues in accordance with the local physiological needs for fatty acids. In agreement with observations made in rodents [[10], [11], [12], [13], [14]], studies in human volunteers have shown that LPL activity in adipose tissue is reduced by fasting [[50], [51], [52], [53], [54]], thereby diverting circulating triglycerides to other tissues. An important and previously unaddressed question was whether the fasting-induced decrease in adipose LPL activity in humans is driven by corresponding changes in LPL expression or whether it is mainly due to a post-translational mechanism via ANGPTL4. Biochemical studies combined with studies in mice have shown that ANGPTL4 is upregulated by fasting in mouse adipose tissue [14,26,32,35] and promotes the unfolding of LPL [33], thereby activating the intracellular cleavage and subsequent degradation of LPL [34,35]. The present data indicate that ANGPTL4 is upregulated by fasting in human adipose tissue, concurrent with a marked decrease in LPL activity and a lack of change in *LPL* mRNA. Although the human data are inevitably correlative, they are highly consistent with conservation of the post-translational control of adipose tissue LPL activity during fasting between rodents and humans via ANGPTL4.

Previously, we showed that ANGPTL4 promotes the degradation of LPL in adipose tissue of mice, thereby reducing the amount of LPL available on the capillary surface [34]. We also found that ANGPTL4 and LPL protein levels negatively correlate in a cross-sectional analysis of human adipose tissue samples from obese individuals [40]. In this study, we observed that the increase in ANGPTL4 protein levels in human adipose tissue upon fasting was not paralleled by a significant decrease in LPL protein. A number of possibilities may explain these findings. First, the method of detecting LPL via immunoblot may not be sufficiently precise to pick up small changes in LPL levels. Here, it should be noted that the mean LPL protein level was lower after fasting, but this change did not reach statistical significance. Second, the immunoblot may measure the wrong LPL pool. Here, it would have been useful to be able to distinguish between EndoH-sensitive and EndoH-resistant LPL, which in mouse adipose tissue can be used to differentiate between inactive ER-resident LPL and active LPL in the Golgi and on the cell surface, respectively. However, we were unable to visualize EndoH-sensitive and EndoH-resistant LPL in human adipose tissue. Hence, it is possible that in human adipose tissue, most of the immunoreactive LPL is inactive and in the ER. Third, the timing of sampling of the adipose tissue biopsies in relation to the meal may not have been optimal. Indeed, in mice, the level of LPL protein in adipose tissue is higher in the re-fed state than in the ad libitum fed state [34]. Fourth, it cannot be excluded that in human adipocytes, ANGPTL4 only unfolds LPL and inhibits LPL activity, but does not regulate LPL degradation and LPL protein levels.

A number of factors may contribute to the upregulation of ANGPTL4 mRNA and protein levels in human adipose tissue during fasting. The expression of *ANGPTL4* in mouse or human adipocytes is known to be regulated via several different signals, including hypoxia (stimulatory), insulin (inhibitory) [35,46], glucocorticoids (stimulatory) [55], tumor necrosis factor α (stimulatory) [56], and PPARγ agonists (stimulatory) [43]. We show that ANGPTL4 levels in human adipocytes are also increased by fatty acids, confirming regulation in other cell types [39,57]. In addition, we find that ANGPTL4 levels in human adipocytes are decreased and increased by insulin and glucocorticoids, respectively. Induction by glucocorticoids is mediated by binding of the glucocorticoid receptor to the 3′-untranslated region of exon 7 [55]. The inhibition of *Angptl4* expression by insulin in mouse adipocytes is likely mediated by the PI3K/Foxo1 pathway [46]. Overall, the data suggest that the increased ANGPTL4 production in adipose tissue upon fasting is likely mediated by changes in the plasma levels of insulin, cortisol, and fatty acids.

Intriguingly, ANGPTL4 was less sensitive to the suppressive effect of insulin in subcutaneous adipocytes than visceral adipocytes. This is in line with our previous finding that ANGPTL4 mRNA levels are higher in subcutaneous adipose tissue than in visceral adipose tissue [40], which in turn is in agreement with the finding that the LPL activity/mass ratio is lower in subcutaneous than visceral adipose tissue [58]. Why insulin less effectively lowers ANGPTL4 in subcutaneous adipocytes is not yet clear.

The clearance of plasma triglycerides is promoted by insulin. Accordingly, the impaired action of insulin in type 2 diabetes leads to reduced plasma triglyceride clearance, which in turn contributes to elevated post-prandial lipid excursions and fasting dyslipidemia [59]. Taking into consideration the repression of adipocyte ANGPTL4 mRNA by insulin [35,46], the upregulation of ANGPTL4 in insulin resistance may contribute to the postprandial dyslipidemia in insulin-resistant individuals via inhibition of LPL. In support, type 2 diabetics present with elevated circulating ANGPTL4 levels [60]. Contradicting this scenario, however, the reduction in adipose ANGPTL4 mRNA during a hyperinsulinemic clamp was similar in insulin-sensitive and resistant individuals. In addition, weight loss, despite an improvement in insulin sensitivity, did not influence ANGPTL4 mRNA levels in human adipose tissue. Existing data on the relation between insulin resistance and adipose tissue LPL activity are mixed as well. In a group of 26 subjects varying in insulin sensitivity, insulin resistance was negatively correlated with adipose tissue LPL activity [61]. Consistent with these data, in type 2 diabetic men, adipose tissue LPL activity was significantly reduced compared to matched non-diabetic subjects, while the differences were more modest in women [62]. By contrast, Olivecrona found that the induction of adipose tissue LPL activity with feeding was similar in type 2 diabetes patients and matched healthy controls, suggesting that dysregulation of adipose LPL is not involved in the postprandial hypertriglyceridaemia in type 2 diabetes [63]. Overall, these data make it difficult to assign a role for aberrant ANGPTL4 regulation in post-prandial hypertriglyceridemia in type 2 diabetes.

We found that human adipose tissue and adipocytes only produce full length ANGPTL4. Based on the inability to detect full length ANGPTL4 in human plasma, it could be reasoned that ANGPTL4 produced in adipose tissue does not end up in the circulation, suggesting that it has a local role. Alternatively, adipose tissue-derived full length ANGPTL4 may undergo cleavage in the circulation. As the plasma concentration of full length ANGPTL4 is probably very low, the ANGPTL4 ELISA, which is able to detect full length and C-terminal ANGPTL4 but not N-terminal ANGPTL4 [45], in essence measures the plasma levels of C-terminal ANGPTL4.

In this paper we find that adipose tissue ANGPTL8 expression is markedly reduced by fasting. Most of the published data relate to the role of ANGPTL8 in the liver, where in the fed state ANGPTL8 forms a complex with ANGPTL3 and supports the inhibition of plasma triglyceride clearance by ANGPTL3 in brown adipose tissue, heart, and muscle, thereby rerouting plasma triglycerides to white adipose tissue for storage [22,24]. Recently, evidence was presented that ANGPTL8, via direct protein interaction, may interfere with the ability of ANGPTL4 to inhibit LPL [36]. Presumably, in the fed state, when ANGPTL8 expression is high, ANGPTL8 suppresses ANGPTL4 function, thereby promoting adipose tissue LPL activity. The extent to which adipose tissue contributes to the changes in plasma ANGPTL8 during fasting is unclear. Interestingly, in human adipocytes, ANGPTL8 mRNA was upregulated by cortisol and dexamethasone, although to a lesser extent than by insulin. The impact of the induction of ANGPTL8 by glucocorticoids on LPL activity needs further investigation.

This paper has limitations. First, our study cannot demonstrate a direct causal link between the upregulation of ANGPTL4 in human adipose tissue during fasting and the decrease in LPL activity. Nevertheless, the plethora of pre-clinical data combined with our data strongly suggest that the upregulation of ANGPTL4, and possibly the downregulation of ANGPTL8, causes the decrease in adipose tissue LPL activity in humans during fasting. Second, we were unable to visualize LPL protein in the human primary adipocytes. For reasons that are unclear, LPL is very hard to detect in primary adipocytes compared to adipose tissue, and its migration is dubious.

In conclusion, our data support the notion that the upregulation of ANGPTL4 mediates the decrease in LPL activity and associated lipid storage in adipose tissue during fasting in humans. The increase in ANGPTL4 production in human adipose tissue by fasting is likely mediated by increased plasma cortisol and free fatty acids, and decreased plasma insulin.

## Author contributions

**Philip M. M. Ruppert** Conceptualization, Methodology, Validation, Formal analysis, Investigation, Writing – Original draft, Writing – Review & Editing, Visualization, Supervision. **Charlotte C.J.R. Michielsen** Conceptualization, Methodology, Formal analysis, Investigation, Resources, Data curation, Writing – Review & Editing, Visualization. **Eric J. Hazebroek** Resources, Writing – Review & Editing. **Ali Pirayesh** Resources, Writing – Review & Editing. **Gunilla Olivecrona** Resources, Writing – Review & Editing. **Lydia A. Afman** Conceptualization, Writing – Review & Editing, Supervision, Project administration, Funding acquisition. **Sander Kersten** Conceptualization, Formal analysis, Writing – Original draft, Writing – Review & Editing, Visualization, Supervision, Project administration, Funding acquisition.
