# Supplementary material for: Fasting induces ANGPTL4 and reduces LPL activity in human adipose tissue
Source: Mol Metab. 2020 Jun 3;40:101033. doi: 10.1016/j.molmet.2020.101033 (PMC7334813; doi:10.1016/j.molmet.2020.101033)
Supplement: Multimedia component 1 [file mmc1.pdf]

Supplementary figures

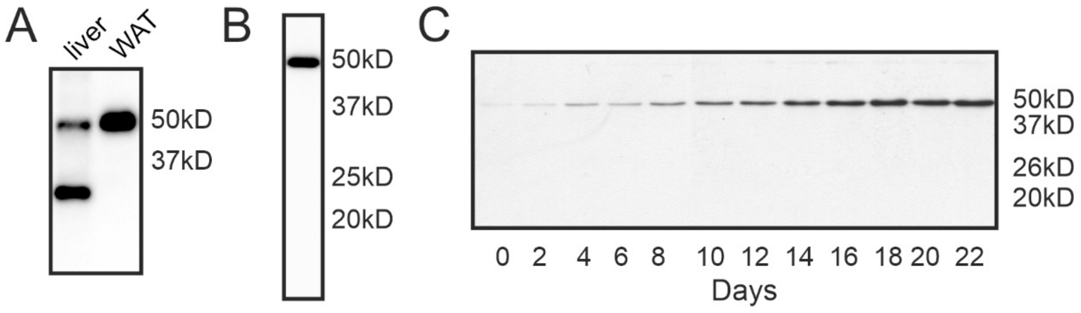

Supplementary figure 1. ANGPTL4 is produced in human adipose tissue as full length protein. A) Immunoblot for ANGPTL4 of human liver and human subcutaneous adipose tissue. B) Immunoblot for ANGPTL4 of human primary adipocytes. C) Immunoblot for ANGPTL4 during adipogenic differentiation of human Lisa-2 adipocytes [1].

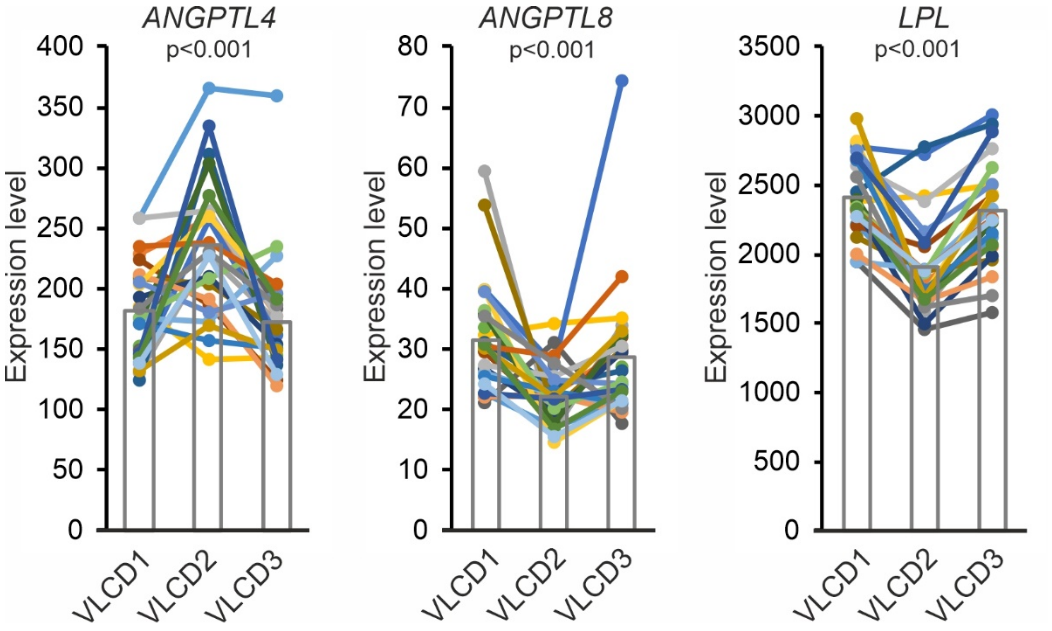

Supplementary figure 2. Adipose tissue mRNA levels of *ANGPTL4*, *ANGPTL8* and *LPL* are altered during severe hypocaloric diet but are unaffected by weight loss per se. Microarray-based gene expression of *ANGPTL4*, *ANGPTL8* and *LPL* in adipose tissue of subjects before weight loss (VLCD1), after 5 weeks of very low calorie diet (500 Kcal/day, VLCD2), and after 4 weeks of weight maintenance (VLCD3) (GSE77962) [2].

Supplementary tables

Supplementary table 1. Primer sequences used for qPCR in this study

**Gene**	**Forward primer**	**Reverse Primer**
ANGPTL4	CACAGCCTGCAGACACAACTC	GGAGGCCAAACTGGCTTTGC
ANGPTL8	CAGAAGGTGCTACGGGACAG	AAATTCTCGGTAGGCAGGGC
LPL	CATTCCCGGAGTAGCAGAGT	GGCCACAAGTTTTGGCACC
BACTIN	AGAAAATCTGGCACCACACC	AGAGGCGTACAGGGATAGCA

References

[1] M. Wabitsch, S. Brüderlein, I. Melzner, M. Braun, G. Mechtersheimer, and P. Möller, "LiSa-2, a novel human liposarcoma cell line with a high capacity for terminal adipose differentiation.," *Int. J. cancer*, vol. 88, no. 6, pp. 889–94, Dec. 2000.

[2] R. G. Vink *et al.*, "Adipose tissue gene expression is differentially regulated with different rates of weight loss in overweight and obese humans," *Int. J. Obes.*, vol. 41, no. 2, pp. 309–316, Feb. 2017.
